# A Systematic Review of Medication Adherence Thresholds Dependent of Clinical Outcomes

**DOI:** 10.3389/fphar.2018.01290

**Published:** 2018-11-20

**Authors:** Pascal C. Baumgartner, R. Brian Haynes, Kurt E. Hersberger, Isabelle Arnet

**Affiliations:** ^1^Pharmaceutical Care Research Group, University of Basel, Basel, Switzerland; ^2^Department of Health Research Methods, Evidence and Impact, McMaster University, Hamilton, ON, Canada

**Keywords:** medication adherence (MeSH), patient compliance, threshold, systematic (literature) review, clinical outcome, adherence measurement methods, adherence metric, adherence methodologies

## Abstract

**Background:** In pharmacotherapy, the achievement of a target clinical outcome requires a certain level of medication intake or adherence. Based on Haynes's early empirical definition of sufficient adherence to antihypertensive medications as taking ≥80% of medication, many researchers used this threshold to distinguish adherent from non-adherent patients. However, we propose that different diseases, medications and patient's characteristics influence the cut-off point of the adherence rate above which the clinical outcome is satisfactory (thereafter medication adherence threshold). Moreover, the assessment of adherence and clinical outcomes may differ greatly and should be taken into consideration. To our knowledge, very few studies have defined adherence rates linked to clinical outcomes. We aimed at investigating medication adherence thresholds in relation to clinical outcomes.

**Method:** We searched for studies that determined the relationship between adherence rates and clinical outcomes in the databases PubMed, Embase^Ⓡ^ and Web of Science™ until December 2017, limited to English-language. Our outcome measure was any threshold value of adherence. The inclusion criteria of the retrieved studies were (1) any measurement of medication adherence, (2) any assessment of clinical outcomes, and (3) any method to define medication adherence thresholds in relation to clinical outcomes. We excluded articles considered as a tutorial. Two authors (PB and IA) independently screened titles and abstracts for relevance, reviewed full-texts, and extracted items. The results of the included studies are presented qualitatively.

**Result:** We analyzed 6 articles that assessed clinical outcomes linked to adherence rates in 7 chronic disease states. Medication adherence was measured with Medication Possession Ratio (MPR, *n* = 3), Proportion of Days Covered (PDC, *n* = 1), both (*n* = 1), or Medication Event Monitoring System (MEMS). Clinical outcomes were event free episodes, hospitalization, cortisone use, reported symptoms and reduction of lipid levels. To find the relationship between the targeted clinical outcome and adherence rates, three studies applied logistic regression and three used survival analysis. Five studies defined adherence thresholds between 46 and 92%. One study confirmed the 80% threshold as valid to distinguish adherent from non-adherent patients.

**Conclusion:** The analyzed studies were highly heterogeneous, predominantly concerning methods of calculating adherence. We could not compare studies quantitatively, mostly because adherence rates could not be standardized. Therefore, we cannot reject or confirm the validity of the historical 80% threshold. Nevertheless, the 80% threshold was clearly questioned as a general standard.

## Introduction

With pharmacotherapy, the achievement of the targeted clinical outcome (e.g., control of high blood pressure or HIV viral load suppression) requires a certain level of medication intake or adherence (Maggiolo et al., 2007; Jung et al., 2013). Adherence to medication is defined as “the extent to which a patient's behavior matches the agreed recommendations from a healthcare provider”(Sabaté, 2003). Individual patient's adherence is usually reported as percentage of the actual medication taken over a defined period of time (i.e., adherence rate) and varies from 0% to over 100% in literature (DiMatteo, 2004; Briesacher et al., 2008; Fischer et al., 2010; Nieuwlaat et al., 2014; Huurne et al., 2015). By using a threshold, patients can be dichotomized in persons who take their medications as prescribed (i.e., adherers) and those who deviate from the recommendations in any way (i.e., non-adherers). Based on Haynes's early empirical definition of sufficient adherence to antihypertensive medications as taking ≥80% of medication (Haynes et al., 1980), many researchers used this threshold to distinguish adherent from non-adherent patients (Caro et al., 2004; Doro et al., 2005; Hansen et al., 2010). In Haynes's study, the 80% threshold was supported by a regression analysis indicating that diastolic blood-pressure only fell systematically above this level of adherence. Unsurprisingly, in most other studies the 80% threshold has been used with no clinical rationale (Steiner and Prochazka, 1997; Doro et al., 2005; Hansen et al., 2010). The misconception of using 80% as universal threshold for good adherence is one remaining myth in 40 years of adherence science (Gellad et al., 2017). We propose that the disease, medication and patient's characteristics influence the cut-off point of the adherence rate above which the clinical outcome is satisfactory (thereafter medication adherence threshold). Moreover, the assessment of adherence and clinical outcomes may differ greatly and should be taken into consideration. Some recent theoretical approaches exist to determine adherence thresholds with computer models such as using simulated pharmacodynamic and pharmacokinetic parameters of statins to simulate the adherence rate needed to reach a LDL-C value below 70 mg/dL (Stauffer et al., 2017). However, to our knowledge, very few studies have defined adherence thresholds according to clinical outcomes. We aimed at defining medication adherence thresholds in relation to clinical outcomes.

## Methods

We searched for studies that determined medication adherence thresholds in relation to clinical outcomes. We conducted a systematic literature search according to the Preferred Reporting Items for Systematic Reviews and Meta-analyses (PRISMA) Guidelines (Moher et al., 2009).

### Eligibility criteria

To be included, a study had to describe (1) any measurement of medication adherence, (2) any assessment of clinical outcomes, and (3) any method to define medication adherence thresholds in relation to clinical outcomes. Citations of the type book chapter, conference proceedings, and dissertations were excluded. We excluded articles considered as a tutorial. We deliberately avoided to restrict our search to a target population, disease, or medication because of the universality of adherence behavior.

### Search strategy and information sources

We developed our strategy utilizing the terms “adherence” and synonyms, and “threshold” and synonyms in the title of publications. The databases PubMed, Embase^Ⓡ^ and Web of Science™ were searched covering the time period from inception to 31st December 2017, limiting to English-language publications. The search strategy for each database is shown in Supplementary Material.

### Study selection

After we removed all duplications, the retrieved citations were screened based on the title and abstract, then on the full text. Two investigators assessed eligibility (PB, IA). Any disparity was resolved by consensus. All work was performed in Endnote™ (Clarivate Analytics, Version X8).

### Data collecting process

Data extraction was performed by one investigator (PB) and a second investigator (IA) checked the worktable for completeness and accuracy. Disagreements were resolved by consensus.

### Data items

We collected the following variables in the included studies: disease; medication class or medication; population; medication adherence measurement; clinical outcomes; study design; method for threshold determination.

### Summary measure

Measures of interest were: mean medication adherence rate with standard deviation; medication adherence threshold value; probability to reach the targeted clinical outcome with the medication adherence threshold (expressed as odds ratio or hazard ratio); percentage of patients below the threshold.

## Result

### Study selection

**Figure 1:**
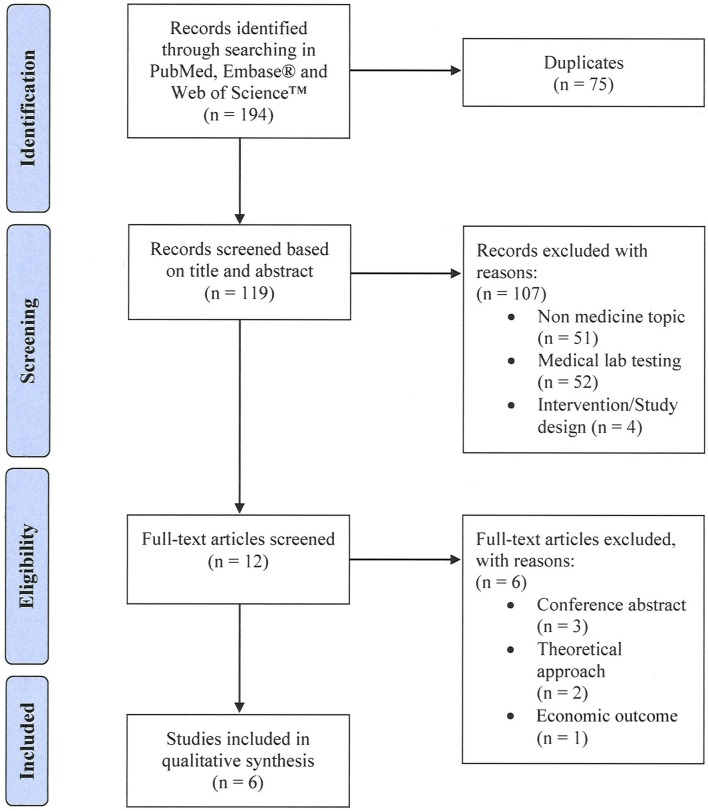
Study flow diagram.

The systematic literature search yielded 194 records. After removal of duplicates, 119 unique citations were screened based on title and abstract. We excluded 107 articles that were not in the field of medicine (*n* = 51), investigated medical lab testing (*n* = 52), or were discussing adherence interventions (*n* = 4). Of the remaining twelve articles that were assessed for eligibility in full text, 6 articles were excluded [conference abstracts (*n* = 3), focusing on economic outcome (*n* = 1), discussing a theoretical approach (*n* = 2)]. Six articles met all set eligibility criteria and were included in our qualitative synthesis (see Figure 1).

### Study characteristics

The 6 identified studies were published between 2009 (Karve et al., 2009; Wu et al., 2009) and 2017 (Govani et al., 2017) and were all conducted in the USA (Karve et al., 2009; Wu et al., 2009; Oleen-Burkey et al., 2011; Watanabe et al., 2013; Lo-Ciganic et al., 2015; Govani et al., 2017). Data originated from insurance services covering patients throughout the USA (Oleen-Burkey et al., 2011; Govani et al., 2017), from a Medicaid program of a state (Karve et al., 2009; Lo-Ciganic et al., 2015), the Department of Veterans affairs (Watanabe et al., 2013) or cardiology clinics in Central Kentucky (Wu et al., 2009). Average age of patients ranged from 41 (Govani et al., 2017) to 68.4 years (Karve et al., 2009), the percentage of females 4.6 (Watanabe et al., 2013) to 81.29% (Oleen-Burkey et al., 2011). The study population ranged from 135 (Wu et al., 2009) to 37,912 patients (Karve et al., 2009). Fives studies (Wu et al., 2009; Oleen-Burkey et al., 2011; Watanabe et al., 2013; Lo-Ciganic et al., 2015; Govani et al., 2017) were focusing on a single chronic disease, while one (Karve et al., 2009) included patients with one out of five chronic disease states (schizophrenia, diabetes, hypertension, hyperlipidemia, and congestive heart failure). Medication adherence was calculated to a single medication in five studies (Karve et al., 2009; Wu et al., 2009; Oleen-Burkey et al., 2011; Watanabe et al., 2013; Govani et al., 2017), or to all hypoglycemic agents in one study (Lo-Ciganic et al., 2015). Two studies (Oleen-Burkey et al., 2011; Watanabe et al., 2013) focused on new medication users, and four studies (Karve et al., 2009; Wu et al., 2009; Lo-Ciganic et al., 2015; Govani et al., 2017) included patients on the medication of interest without further explanations.

### Study design

Five studies were retrospective with pharmacy claims data (Karve et al., 2009; Oleen-Burkey et al., 2011; Watanabe et al., 2013; Lo-Ciganic et al., 2015; Govani et al., 2017), one study was designed as a prospective study using an electronic medication bottle (MEMS^Ⓡ^) (Wu et al., 2009). The observation period ranged from 1 (Karve et al., 2009; Watanabe et al., 2013; Lo-Ciganic et al., 2015) to 4 years (Govani et al., 2017). Three studies observed adherence and clinical outcome simultaneously (Oleen-Burkey et al., 2011; Watanabe et al., 2013; Govani et al., 2017) while three studies assessed sequentially first adherence, followed by the targeted clinical outcome (Karve et al., 2009; Wu et al., 2009; Lo-Ciganic et al., 2015). The period during which medication adherence was measured ranged from 3 months (Wu et al., 2009) to 4 years (Govani et al., 2017). The occurrence of the targeted clinical outcome was assessed over 1 year (Karve et al., 2009; Watanabe et al., 2013; Lo-Ciganic et al., 2015) up to 4 years (Govani et al., 2017).

### Medication adherence measures

Retrospective database studies measured adherence by calculating the Medication Possession Ratio (MPR; this measure assesses the proportion of time with adequate supply over a predefined observation period) (Oleen-Burkey et al., 2011; Watanabe et al., 2013; Govani et al., 2017), or the Proportion of Days Covered (PDC; this measure represents the proportion of days a patient has a medication available in a given period of time, mostly a calendar year) (Lo-Ciganic et al., 2015) or both (Karve et al., 2009). Different definitions and operationalization of the MPR and PDC were used (See Table 1). In the MEMS^Ⓡ^ study (Wu et al., 2009), adherence rates were defined as the percentage of prescribed doses taken (dose count) and percentage of days with correct number of doses taken (dose day). Adherence outliers were truncated at 100 (Karve et al., 2009; Lo-Ciganic et al., 2015), at 140% (Govani et al., 2017), or not (Wu et al., 2009; Oleen-Burkey et al., 2011; Watanabe et al., 2013).

**Table 1:** Characteristics of included studies.

**Study [Ref] (year)**	**Disease**	**Medication class**	**Population**				**Study design**		**Adherence measure [verbatim]**	**Outcome measure**	**Threshold determination**
		**(specific medication)**	**Data sources (State/Country)**	***n* = sample size**	**Age ± s.d. [years]**	**Female [%]**	**Inclusion period DD/MM/JJJJ**	**Order of observation:adherence $\overset{}{\underset{}{\to }}$ outcome $\;--\overset{}{\underset{}{\to }}$**		
Govani et al., 2017	Inflammatory bowel disease	Biologicals (Adalimumab: ADA Certolizumab: CZP)	Truven Health MarketScan Commercial Claims and Endcounters database (–/USA)	Overall *n* = 6'048	41.0 ± 15.0	54.0	01/01/2009–31/12/2013	$\overset{4\text{years}}{\overset{}{\underset{}{\to }}}$ $\overset{4\text{years}}{---\to }$	[-1pt]MPR = $\frac{\text{[n] days of medication}}{\text{[n] of days in the total refill intervals}}$ MPR capped at 1.4	Any disease flare defined as: hospital-ization; or new corticosteroid use	**Survival analysis**: According the Log-Rank: Contal and O'Quigley Method
				ADA *n* = 5'325	41.3 ± 15.3	53.8				
				CZP *n* = 723	41.1 ± 13.8	57.8				
Karve et al., 2009	Schizophrenia	–	Medicaid administrative claims data (Arkansas/USA)	*n* = 3'395	42.9 ± 13.2	75.7	01/07/2000–31/12/2004	$\overset{1\text{year}}{\overset{}{\underset{}{\to }}}\begin{array}{c} ▪\\ ▪\\ ▪\\ ▪\\ ▪\\ ▪\\ ▪ \end{array}$ $\overset{1\text{year}}{-\to }$	[4pt]MPR = $\frac{\text{[n] of days supplied in the index year}}{\text{[n] of days patient is ambulatory}}$ PDC = MPR capped at 1.0	[3pt]Any-cause and disease-related hospitalization	**Regression analysis**: Logistic regression C-statistic based on ROC model
	Diabetes	–		*n* = 4'943	60.9 ± 15.9	74.4				
	Hypertension	–		*n* = 16'398	59.6 ± 17.5	73.8				
	Hyperlipidemia	–		*n* = 7'925	59.6 ± 14.0	79.2				
	Congestive heart failure (CHF)	–		*n* = 5'251	68.4 ± 15.7	75.7				
Lo-Ciganic et al., 2015	Diabetes type II	All oral hypoglycemic medications	Medicaid administrative claims data (Pennsylvania/USA)	n = 33'130	48.3 ± 10.0	66.5	01/07/2007–31/12/2009	$\overset{1\text{year}}{\overset{}{\underset{}{\to }}}\begin{array}{c} ▪\\ ▪\\ ▪\\ ▪\\ ▪\\ ▪\\ ▪ \end{array}$ $\overset{1\text{year}}{-\to }$	PDC = $\frac{\text{[n] of days of medication supplied}}{\begin{array}{c} \text{[n] of days between first and last prescription}+\\ \text{accumulated [n] days of last prescription} \end{array}}$ PDC truncated at 1.0	Time to first all-cause hospitalization	**Survival analysis**: Survival trees and random survival forests
Oleen-Burkey et al., 2011	Multiple sclerosis	Initial use Copaxone (Glatiramer acetate)	i3 Invision™ Data Mart (–/USA)	*n* = 839	45.17 ± 10.4	81.3	01/07/2006–01/04/2008	$\overset{2\text{year}}{\overset{}{\underset{}{\to }}}$ $\overset{\text{2 year}}{--\overset{}{\underset{}{\to }}}$	MPR = $\frac{\text{[n] of days supply glatiramer available}}{\text{[n] of days in the post period}}$ All injection also in physicians office or hospital	Relapse defined as hospitalization/or corticosteroid prescription	**Regression analysis** Logistic regression
Watanabe et al., 2013	–	Statins	Department of Veteran Affairs (VA), (California, Nevada/USA)	*n* = 4'691	63.3 ± 10.9	4.6	30/11/2006–02/12/2007	$\overset{1\text{year}}{\overset{}{\underset{}{\to }}}$ $\overset{\text{1 year}}{--\overset{}{\underset{}{\to }}}$	MPR = $\frac{\text{[n] days' supply of medication dispensed}}{\text{[n] of days based on the prescriptions}}$	25% or more reduction of lipid levels: non-high density lipoprotein (non-HDL) cholesterol; low density lipoprotein (LDL); cholesterol total cholesterol (TC)	**Regression analysis**: Multiple logistic regression Cochran-Armitage trend test
Wu et al., 2009	Heart failure (HF)	Beta blocker/ACE-inhibitor Angiotensin receptor blocker/ Digoxin	Cardiology clinics (Central Kentucky/USA)	*n* = 135	61.0 ± 11.0	30.4	Not reported	3 months 3.5 years $\overset{}{\underset{}{\to }}\begin{array}{c} ▪\\ ▪\\ ▪\\ ▪\\ ▪\\ ▪\\ ▪ \end{array}$ −− →	Medication event monitoring system (MEMS^Ⓡ^) dose count: percentage of prescribed doses taken dose days: percentage of days with correct number of doses taken	Event free survival; event defined as: symptoms of decompensated HF; or cardiac rehospitalization; or mortality	**Survival analysis**: Log-Rank Kaplan-Meier Cox-survival analysis, ROC model

### Clinical outcomes

Five studies used event free survival as clinical outcome (Karve et al., 2009; Wu et al., 2009; Oleen-Burkey et al., 2011; Lo-Ciganic et al., 2015; Govani et al., 2017) and one study used the reduction of lipid levels (Watanabe et al., 2013). Events were defined as mortality (Wu et al., 2009), hospitalization (Karve et al., 2009; Wu et al., 2009; Oleen-Burkey et al., 2011; Lo-Ciganic et al., 2015; Govani et al., 2017), cortisone use (Govani et al., 2017), cortisone prescription (Oleen-Burkey et al., 2011), and reported symptoms (Wu et al., 2009). Studies using hospitalization as clinical outcome were either including all-cause hospitalization (Oleen-Burkey et al., 2011; Lo-Ciganic et al., 2015; Govani et al., 2017), disease specific hospitalization (Wu et al., 2009) or both (Karve et al., 2009).

### Threshold determination

Two methods were applied to link the targeted clinical outcome and adherence rates: logistic regression [i.e., correlating the independent variable “adherence” with the dependent dichotomized variable “outcome” (Karve et al., 2009; Oleen-Burkey et al., 2011; Watanabe et al., 2013)] and survival analysis [i.e., comparing different adherence rate groups in regard to time to event rates (Wu et al., 2009; Lo-Ciganic et al., 2015; Govani et al., 2017)]. Studies using logistic regression determined the optimal threshold based on Receiver Operating Characteristic curve (i.e., a method that plots sensitivity/specificity values to a particular decision threshold) (Karve et al., 2009); or compared the odds ratio of different adherence rate groups for a relapse (Oleen-Burkey et al., 2011) or for achieving a therapeutic goal (Watanabe et al., 2013). For survival analysis, maximized log rank statistics generated two adherence groups that separated most significantly either by shifting the threshold and comparing the resulting dichotomized adherence groups (Wu et al., 2009) or using a macro (Contal and O'Quigley, 1999) that calculates log rank statistics for all possible thresholds (Govani et al., 2017) or a special approach developing a random survival forest model for predictor of hospitalization with adherence being one of fifteen predictors for hospitalization (Lo-Ciganic et al., 2015).

### Adherence thresholds and clinical outcomes

Four studies reported mean adherence rates (Karve et al., 2009; Lo-Ciganic et al., 2015; Govani et al., 2017) between 61% in congestive heart failure (Karve et al., 2009) and 94% in patients with inflammatory bowel disease (Govani et al., 2017). Adherence rate thresholds linked to the targeted clinical outcome ranged from 63% for congestive heart failure (Karve et al., 2009) to 90% for statins (Watanabe et al., 2013). In the study with diabetes type II, threshold values were determined depending on other predictors of hospitalization (such as prior hospitalization, number of monthly prescriptions, insulin use), and ranged from 46 to 92% (Lo-Ciganic et al., 2015). In the retrieved studies, the relationships between the medication adherence thresholds and the clinical outcomes were expressed as odds ratio (Karve et al., 2009; Oleen-Burkey et al., 2011; Watanabe et al., 2013) or hazard ratio (Wu et al., 2009; Lo-Ciganic et al., 2015; Govani et al., 2017). For example, the hazard ratio for a flare was 0.75 for patients achieving an MPR of 0.86 (i.e., patients who reached a Medication Possession Ratio of 86% had a 25% lower risk to have a flare) (Govani et al., 2017). For all values, see Table 2.

**Table 2:** Summarized results of the included studies.

**Study [Ref] (year)**	**Disease or medication class**	**Mean medication adherence rate ± standard deviation**	**Medication adherence rate threshold**	**Probability to reach the targeted clinical outcome with the medication adherence threshold (odds ratio [OR], hazard ratio [HR], and confidence interval [CI])**	**Percentage of patients below medication adherence threshold**
Govani et al., 2017	Adalimumab	MPR 0.94 ± 0.13	**0.86**	Hazard Ratio (HR): 0.75 (95% CI 0.67–0.83) for a flare	24%
	Certolizumab	MPR 0.87 ± 0.14	**0.87**	HR: 0.59 (95% CI 0.46–0.76) for a flare	24%
Karve et al., 2009	Schizophrenia	MPR 0.738 ± 0.310	**0.76**	OR: 0.456 for disease related hospitalization	–
		PDC 0.724 ± 0.295	**0.76**	OR: 0.430 for disease related hospitalization	–
	Diabetes	MPR 0.763 ± 0.279	**0.85**	OR: 0.449 for disease related hospitalization	–
		PDC 0.751 ± 0.266	**0.85**	OR: 0.434 for disease related hospitalization	–
	Hypertension	MPR 0.712 ± 0.304	**0.82**	OR: 0.712 for disease related hospitalization	–
		PDC 0.702 ± 0.293	**0.82**	OR: 0.708 for disease related hospitalization	–
	Hyperlipidemia	MPR 0.731 ± 0.295	**0.81**	OR: 0.591 for disease related hospitalization	–
		PDC 0.722 ± 0.284	**0.81**	OR: 0.581 for disease related hospitalization	–
	Congestive heart failure	MPR 0.619 ± 0.304	**0.58**	OR: 0.856 for disease related hospitalization	–
		PDC 0.612 ± 0.295	**0.58**	OR: 0.855 for disease related hospitalization	–
Lo-Ciganic et al., 2015	Diabetes type II	0.65 ± 0.26	**0.46–0.94**	HR: 0.48-0.69 for all cause hospitalization according the patient health and medication complexity	–
Oleen-Burkey et al., 2011	Multiple sclerosis	–	**0.7**	OR: 0.547 (95% CI 0.362–0.826) for relapse	49.23%
Watanabe et al., 2013	Statins	–	**0.9**	OR: 12.90 (95% CI 9.60–17.35) for 25% reduction of non-HDL cholesterol	–
				OR: 11.29 (95% CI 8.61–14.80) for 25% reduction of LDL cholesterol	–
				OR: 9.11 (95% CI 6.62–12.53) for 25% reduction of total cholesterol	–
Wu et al., 2009	Heart failure	Dose count: 0.887 ± 0.156	**0.88**	HR: 2.2 for time to first event for the non-adherent group	44%
		Dose day: 0.808 ± 0.228	**0.88**	HR: 3.2 for time to first event for the non-adherent group	44%

## Discussion

To our knowledge, this is the first systematic review that aimed at defining medication adherence threshold in relationship to a targeted clinical outcome, and shed light on the historical 80% threshold. Six studies published in the past 9 years met our eligibility criteria and demonstrate the low interest in the question or the complexity of the task. Five studies critically questioned the commonly used 80% adherence threshold as being suboptimal. However, studies were highly heterogeneous predominately concerning study design, clinical outcomes, number of included patients and underlying diseases. Further, various methods exist for the assessment of medication adherence and for its calculation, according to the research setting. Therefore, we were unable to standardize the adherence rates of the different measures, and could not compare the included studies quantitatively. A general agreement to reject or confirm the historical 80% threshold cannot be given due to the low number and the high diversity of the included studies. However, we could summarize some findings to guide future research.

### Medications under investigation

Three studies investigated one medication as surrogate for multiple treatments in the disease of interest (Karve et al., 2009; Wu et al., 2009; Watanabe et al., 2013). This was done with the rationale that a single medication suffices to detect the medication intake behaviors of a patient. However, medication adherence is known to be negatively influenced by a large number of medications or the complexity of treatment (Marcum and Gellad, 2012). In diseases with simple or limited drug regimens, such as hyperlipidemia or multiple sclerosis, it is possible to choose medications as a surrogate with similar properties out of a chemical subgroup [such as HMG-CoA reductase inhibitors (Watanabe et al., 2013)] or even special chemical substance [such as glatiramer acetate (Oleen-Burkey et al., 2011)]. In progressive diseases complex drug regimens are common. As for example, according to the European Society Cardiology (ESC) guidelines, treatments for congestive heart failure (Ponikowski et al., 2016) consist of up to four simultaneous medications with different mechanisms of action. Thus, selecting one single medication as a surrogate for a complex treatment needs clear ground, especially when adherence parameters will be extrapolated from a lead medication to the entire regimen. Therefore, we recommend to include all concerned medications when investigating the intake behaviors of a patient.

### Clinical outcome and observation period

Ideally, there are two types of outcome markers available for analysis, intermediary outcomes (surrogate measures such as blood pressure, lipids, glucose), and patient-important outcomes [e.g., death, stroke, myocardial infarction, hospitalization (Yordanov et al., 2018)]. The latter would require much larger and longer studies—but they would answer the key question of whether the adherence level makes a clinically important difference. Five studies (Karve et al., 2009; Oleen-Burkey et al., 2011; Watanabe et al., 2013; Lo-Ciganic et al., 2015; Govani et al., 2017) used hospitalization as outcome marker for a various diseases such as diabetes (Wu et al., 2009), congestive heart failure (Wu et al., 2009), schizophrenia (Karve et al., 2009), and hyperlipidemia (Watanabe et al., 2013); and for various medications such as adalimumab (Govani et al., 2017) and galtiramer acetate (Oleen-Burkey et al., 2011). Surrogate markers were seldom described (Watanabe et al., 2013). However, the observation periods were mostly 1 year (Karve et al., 2009; Watanabe et al., 2013; Lo-Ciganic et al., 2015), which is short to observe hard endpoints such as hospitalization. Even if hospitalization is easy to document and allows dichotomization for statistical analysis, many cofactors influence the probability of hospitalization in a year such as number of monthly prescriptions, prior hospitalization and disease severity (Lo-Ciganic et al., 2015). As a comparison, the follow-up period of randomized controlled trials with statin therapy and patient important outcome measures (major coronary event, stroke, death) was at least 3 years (Cheung et al., 2004). Consequently, for smaller studies with short observation periods, fast reacting surrogate measures such as blood pressure seem more suitable endpoints to link adherence level with single medication. Thus, researchers should select a specific clinical endpoint and an observation period long enough to catch the full effect of medication adherence on the target clinical outcome.

### Calculation of medication adherence

Even without a gold standard (Lam and Fresco, 2015), any mathematical method used to compute medication adherence needs to be clearly defined (Arnet et al., 2016). Many studies demonstrated that medication possession ratio (MPR) is highly influenced by the observation period (Kozma et al., 2013; Sperber et al., 2017) and oversupply (Martin et al., 2009). Thus, it is surprising that the four retrieved studies that used MPR (Karve et al., 2009; Oleen-Burkey et al., 2011; Watanabe et al., 2013; Govani et al., 2017) present four different formulas with poor specification. Consequently, each study is a standalone and direct comparison is impossible.

Further, according to a new adherence taxonomy (Vrijens et al., 2012), behaviors differ whether patients are initiating, implementing, or discontinuing their treatment. Calculating adherence rate from claims data delivers an aggregate estimate of a patient's medication possession. MPR and PDC are summary measures and cannot differentiate between implementation and discontinuation, mainly because pharmacy claims data do not allow to define precisely the time point of discontinuation. Currently, no method to calculate medication adherence from claims data seems adequate to deliver values for each phase of medication adherence. Researchers need to be aware of the prerequisites of the calculation measure they plan to use.

### Dichotomizing continuous data

To determine medication adherence thresholds, the authors of the studies categorized the population in two groups that vary significantly. Dichotomizing is commonly used in medicine, because it makes data summarization more efficient and offers a simple risk classification in populations for clinicians. However, statisticians advise against dichotomizing continuous data such as medication adherence data, because a substantial loss of information can occur (Streiner, 2002; Royston et al., 2006). Further, replicates of thresholds are made impossible in subsequent studies. As a consequence, the continuous variable “medication adherence” should be described with a distribution plot to present the entire data. In the retrieved studies, only mean adherence value and standard deviation (as indicator of homogeneity) were given to describe the data (Karve et al., 2009; Wu et al., 2009; Lo-Ciganic et al., 2015; Govani et al., 2017). These two values are insufficient to describe the distribution of the data. A graphic such as a histogram of the medication adherence values could deliver additional and comprehensive information covering the distribution.

### Adherence threshold in context of the clinical relevance

The novelty of the retrieved studies was not to try to distinguish adherent from non-adherent patients, but to express the clinical benefit obtained by patients reaching a certain level of engagement in their dosing regimens. Thus, categorizing patients in arbitrary groups such as “good” and “poor” adherer is misleading. On the contrary, to indicate the degree of execution of a treatment in form of a medication adherence threshold represents valuable and concrete information for clinicians. Surprisingly, only half of the studies (Wu et al., 2009; Oleen-Burkey et al., 2011; Govani et al., 2017) presented the percentage of the population below their medication adherence threshold. Thus, this information combined with the adherence distribution (mean value, standard deviation, graphic representation) should enable healthcare providers and policy makers to target patients with low adherence that would clearly clinically benefit reaching a certain level of adherence.

### Limitations

We acknowledge some limitations. First, we may have missed articles that did not contain our search words in their title. However, it is likely that such articles have mentioned adherence threshold in a subsidiary content and then would not have filled our inclusion criteria. For example, a recently published study investigating the adherence to antihypertensive medications and the risk of cardiovascular disease among older adults did not define an unambiguous threshold (Yang et al., 2017). Second, the search was limited to English-language. Third, all included studies were performed in the US-population with inherent specificities such as the underrepresentation of women [US veterans with a percentage 4.6% women (Watanabe et al., 2013)], population with lower income patients [Medicaid enrollees (Karve et al., 2009; Lo-Ciganic et al., 2015)] or a small locally defined population (Wu et al., 2009). Consequently, our results cannot be generalized to other populations. Fourth, due to the diversity of studies, the quantitative comparison of adherence thresholds was not possible.

## Conclusions

This study revealed a large research gap in determining medication adherence thresholds in relationship to clinical outcomes. The authors of the included studies must be complimented for their attempt to question the historical 80% threshold. We were able to extract five recommendations for future research in this field:

1. Include all medications prescribed for a disease to estimate the medication intake behavior;
2. Select an observation period sufficiently long to detect the targeted clinical outcome; orientate to the length of the observation period used in high quality studies;
3. Define the adherence measurement; calculations have to be replicable;
4. Select statistical methods for the threshold determination carefully, in order to avoid loss of information;
5. Put the adherence threshold in context to clinical relevance.

Based on this new knowledge, further studies are needed to define adherence thresholds linked to the targeted clinical outcome in order to deliver high quality and comparable results to ultimately guide healthcare professionals.

## Author contributions

PB designed the review protocol, carried out the literature search, extracted data from selected studies, and drafted the manuscript. IA participated in the literature search. RH, KH, and IA revised the manuscript critically for intellectual content. All authors read and approved the final manuscript.

### Conflict of interest statement

The authors declare that the research was conducted in the absence of any commercial or financial relationships that could be construed as a potential conflict of interest.
