# Supplementary material for: A Systematic Review of Medication Adherence Thresholds Dependent of Clinical Outcomes
Source: Front Pharmacol. 2018 Nov 20;9:1290. doi: 10.3389/fphar.2018.01290 (PMC6256123; doi:10.3389/fphar.2018.01290)
Supplement: Supplementary file 1 [file Table_1.DOCX]

| **PubMed** |
| --- |
| (((((Adherence[ti]) OR compliance[ti] OR medication[ti])) AND (((((((cut$point*[ti]) OR cut$off*[ti]) OR boundary*[ti]) OR threshold*[ti]) OR set$point*[ti]) OR reference value*[ti]) OR limit value*[ti]))) Filters: Publication date from 1900/01/01 to 2017/12/31; English |
| **Embase®** |
| (adherence:ti OR compliance:ti OR medication:ti) AND (cutpoint:ti OR cutoff:ti OR boundary:ti OR threshold:ti OR setpoint:ti OR reference*value:ti OR limit*value:ti) AND [english]/lim AND [<1966-2018]/py |
| **Web of Science™** |
| (TI=(adherence OR compliance OR medication)) AND (TI = (cutpoint OR cutoff OR boundary OR threshold OR setpoint OR reference value OR limit value)) |
